# Astroglial Dysfunctions in Mood Disorders and Rodent Stress Models: Consequences on Behavior and Potential as Treatment Target

**DOI:** 10.3390/ijms25126357

**Published:** 2024-06-08

**Authors:** Yashika Bansal, Sierra A. Codeluppi, Mounira Banasr

**Affiliations:** 1 Campbell Family Mental Health Research Institute, Centre for Addiction and Mental Health (CAMH), Toronto, ON M5T 1R8, Canada; 2 Department of Pharmacology and Toxicology, University of Toronto, Toronto, ON M5G 2C8, Canada; 3 Department of Psychiatry, University of Toronto, Toronto, ON M2J 4A6, Canada

**Keywords:** astroglia, depression, post-traumatic stress disorder, chronic stress, anxiety, anhedonia

## Abstract

Astrocyte dysfunctions have been consistently observed in patients affected with depression and other psychiatric illnesses. Although over the years our understanding of these changes, their origin, and their consequences on behavior and neuronal function has deepened, many aspects of the role of astroglial dysfunction in major depressive disorder (MDD) and post-traumatic stress disorder (PTSD) remain unknown. In this review, we summarize the known astroglial dysfunctions associated with MDD and PTSD, highlight the impact of chronic stress on specific astroglial functions, and how astroglial dysfunctions are implicated in the expression of depressive- and anxiety-like behaviors, focusing on behavioral consequences of astroglial manipulation on emotion-related and fear-learning behaviors. We also offer a glance at potential astroglial functions that can be targeted for potential antidepressant treatment.

## 1. Introduction

**Figure 1:**
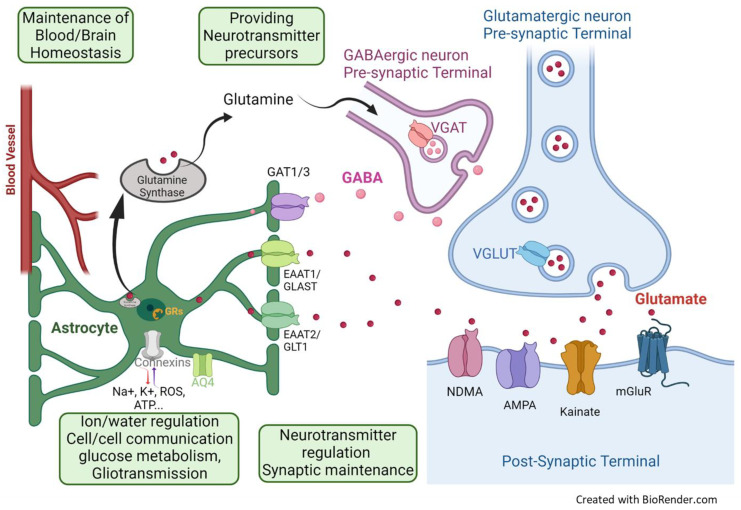
Key functions of astroglial cells. Astroglia play an important role in synaptic maintenance, reuptake of neurotransmitters from the post-synaptic space, recycling of GABA and glutamate, cell–cell communication, the formation of the perivascular space, etc. In this figure, we highlight the select functions and molecules we mention in the review. Abbreviations: AMPA receptor: α-amino-3-hydroxy-5-methyl-4-isoxazolepropionic acid receptor, AQ4: aquaporin 4, ATP: adenosine triphosphate, EAAT 2: excitatory amino acid transporter 2 in human or GLT1: glial glutamate transporter 1 in rodent, EAAT1: excitatory amino acid transporter 1 in human or GLAST: L-glutamate/L-aspartate transporter 1 in rodent, GABA: gamma-aminobutyric acid, GR: glucocorticoid receptor, mGluR: metabotropic glutamate receptor, NMDA receptor: N-methyl-D-aspartate receptor, ROS: reactive oxygen species, VGAT: vesicular GABA transporter.

Astroglia cells are the most populous glial subtype in the CNS. They are named for their star-shaped appearance consisting of a central soma with numerous protruding perisynaptic astrocytic processes and end feet that contact blood vessels [1,2]. Before the turn of the 19th century, astroglia was considered a homogenous cell type that performed a supportive and structural “glue” role in the CNS [3]. However, it is now understood that astrocytes are an extremely heterogeneous population of cells that have diverse roles, including blood/brain homeostatic maintenance, ion–water homeostasis, synapse development, and neurotransmitter metabolism and regulation of neurotransmission as part of the tripartite synapse [4] (Figure 1). Indeed, within a brain region and between brain regions, this class of neuroglial cells are highly heterogeneous in morphology and are unified by their common origin and functions [5,6,7]. The astroglial functions and subtypes can vary significantly based on the size, morphology, and transcriptomic profile. However, they maintain ~850 consistent genes, with functions related to metabolism, cholesterol biosynthesis, and neurotransmitter uptake and biosynthesis [6,8].

Astrocytes can generally be divided into two subpopulations: protoplasmic and fibrous. Protoplasmic astrocytes are located throughout the gray matter and have a morphological pattern of uniformly distributed stem branches, with many finely branching processes that envelop synapses [9,10]. They typically express S100 calcium-binding protein B (S100B) and/or glial fibrillary acidic protein (GFAP) [7]. Interestingly, S100B in gray matter is specific to astrocytes, but in white matter, it can also be found in myelinating oligodendrocytes [11]. On the other hand, GFAP is specific to astrocytes [12] and is found in both gray and white matter, with significantly higher levels in white-matter astrocytes [13]. This makes GFAP a more specific marker or promotor (compared to S100B) to investigate or manipulate astroglia for research.

Further defining astroglia into reactive and non-reactive astrocytes has been an area of debate in the study of the astroglial changes associated with psychiatric disorders. It is generally understood that healthy astroglia, regardless of CNS region or population subtype, can become reactive depending on sudden external influences, including stress exposure producing diverse molecular effectors [14,15]. Reactive astrocytes can be further divided into two subtypes: the neurotoxic (A1) and neuroprotective phenotypes (A2) [14]. However, the evidence differentiating A1 and A2 astrocytes is mixed and likely does not encompass the true heterogeneity of reactive astrocytes and requires further research to establish evidence for phenotype-specific markers [14,16]. For this reason, the current research investigating astroglial dysfunctions associated with stress-related illnesses often does not distinguish between these two subgroups. Instead, the focus has mostly been on understanding the overall GFAP- and S100B-astroglia dysfunctions.

Given the diverse role of astroglial cells (Figure 1), it is understandable that their dysfunction can cause havoc in the brain and has been implicated in the pathology of several brain-related illnesses, including psychiatric disorders such as major depressive disorder (MDD) and post-traumatic stress disorder (PTSD) [17,18,19,20,21,22]. Stress is one of the major risk factors and environmental influences in these mood disorders [23]. In this review, we probe the effects of stress on astroglia and delve into astroglial deficits in mood disorders in humans, with a focus on MDD and PTSD. We also expand on the potential mechanisms involved, based on research using chronic stress rodent models. We further summarize the findings, examining the effects of astrocyte manipulations concentrating on studies by specifically investigating the emotion-related behaviors and mechanisms that suggest that targeting astroglia is a valid avenue for treatment.

## 2. Astroglia Are Affected by Stress

Stress is a major factor in the development of mood disorders [23], and chronic exposure increases the allostatic load and challenges the homeostatic/allostatic capability of brain cells [24,25,26]. Since astroglia are critical mediators of brain homeostasis and are one of the first points of contact for many peripheral signals of stress and inflammation, such as corticosteroids and glucocorticoids (GCs), they are highly affected by chronic stress exposure [19,27,28]. To better understand this phenomenon, it is pivotal to first describe the direct effects of stress on the CNS.

The main response to stress occurs via the activation of the hypothalamic–pituitary–adrenal (HPA) axis. During stress conditions, corticotropin-releasing factor (CRF) activates the HPA axis and initiates a cascade of events that culminate in the release of GCs, particularly corticosterone in rodents and cortisol in humans [29,30]. GCs initiate a cellular stress response through binding to GC receptors (GR) and have epigenetic effects on gene transcription [31]. The dysfunction of this GC pathway can increase an individual’s vulnerability to stress [29]. Interestingly, recent work showed that astrocytes are much more sensitive to stress than neurons, as astroglia display higher expression of GRs [32]. The position of the astroglial endfeet at the Virchow–Robin space makes astrocytes a primary bridge between peripheral corticosteroids/GC and the CNS [33], thus highlighting their importance in the mediation of the effects of stress.

Further studies implicating astrocytes as the bridge between stress and stress response have investigated microRNAs (miRNAs) as an avenue for stress regulation [34,35]. miRNAs are short, non-coding, post-transcriptional regulator sequences of RNA that control the expression of protein-coding genes for cellular proliferation, differentiation, immune response, and apoptosis [36]. They are pivotal to stress response, given that they can target multiple transcripts at once. However, in the presence of homeostatic collapse, the biogenesis of miRNA and, therefore, transcription can be disrupted, making the cell more susceptible to stress [35]. These miRNAs are also present in extracellular vesicles (EVs), one avenue for intercellular communication. Interestingly, some miRNA in the EVs secreted by astrocytes are up- or downregulated in stress conditions. This has led to postulations that astrocytic release miRNAs in EVs modulate neurogenesis and synaptogenesis in stress conditions [34]. Indeed, recent work in rodent stress models shows that miRNA in astroglial-released EVSs regulate dendritic complexity [37]. It should be noted that miRNA in astroglial-released EVs can also be potentially neuroprotective in other disease models [38,39,40].

## 3. Astroglial Dysfunction in Mood Disorders

MDD is a severe mental health disorder, with symptoms including feelings of helplessness/worthlessness, and anhedonia, a pivotal symptom of diagnosis [41]. MDD is often comorbid with anxiety symptoms [42,43,44,45]. On the other hand, PTSD is an anxiety disorder with an onset characterized by the presence of a sudden, extreme, traumatic, relatively acute, stressor. PTSD is accompanied by mood symptoms that include negative feelings, loss of interest in pleasurable activities, hyper-arousal, and cognitive dysregulation [41]. Astroglial dysfunctions are reported in both disorders and may be linked to the shared symptomology. In this section, we will outline the extensive findings supporting the critical role of astroglial dysfunction in MDD and the relatively less extensive but relevant research in PTSD.

### 3.1. Evidence of Astroglial Dysfunction in MDD

**Figure 2:**
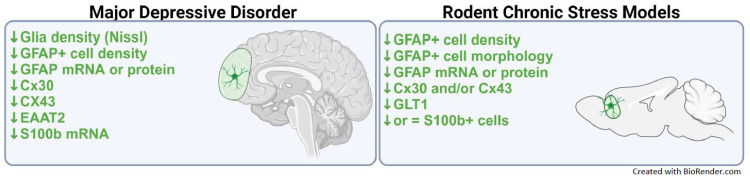
Astroglial dysfunctions in the PFCs of patients with MDD, highlighting similarity with rodent chronic stress models. Abbreviations: EAAT 2: excitatory amino acid transporter 2, GLT1: glial glutamate transporter 1, GFAP: glial fibrillary acidic protein, Cx30 or Cx43: Connexin 30 or 43.

Astroglial dysfunctions in MDD have been consistently reported in human MDD post-mortem patients [18,19,27,46] (Figure 2). The first studies used Nissl staining to identify glia, including astroglia, based on their morphology and described reduction in MDD brains [47,48,49,50]. These findings were then later confirmed using GFAP as an astroglial marker, reporting reductions in GFAP+ astroglia number in the prefrontal cortex (PFC), the white matter of the anterior cingulate cortex (ACC), the hilus region of the hippocampus, and the amygdala in patients with MDD [18,46,48,51,52,53]. However, there are some exceptions. Indeed, some post-mortem MDD studies reported no change in astrocyte density in the orbitofrontal cortex or CA1 or CA2/3 regions of the hippocampus and increased GFAP+ astroglia density in the PFC of MDD patients over 60 years of age [54,55,56]. MDD post-mortem studies have also shown significantly lower GFAP mRNA and protein levels in areas of the PFC [57,58,59,60,61] (Figure 2). In addition to alterations in the GFAP astroglia number or GFAP expression in CNS, patients with MDD also had reduced coverage of blood vessels by astroglial endfeet [62] and increased levels of GFAP protein in the cerebrospinal fluid (CSF) [63]. As GFAP is a cytoarchitectural protein, together, these studies suggest that MDD is associated with GFAP astroglia loss or atrophy in several brain regions.

Changes in the S100B astroglia were also reported in MDD. Indeed, post-mortem studies showed a reduced transcription of S100B in the PFC of MDD suicide completers [46,64] and a reduction in S100B+ cell density in the CA1 region of the hippocampus of MDD brains [65]. Although some studies described peripheral changes in serum S100B in MDD patients, this was not systematically found in MDD or across psychiatric disorders [65,66,67,68]. Although controversial results were reported between MDD severity and plasma/CSF S100B levels in different clinical studies [69,70,71], a meta-analysis showed significantly positive correlations between MDD severity and plasma/CSF S100B levels [67]. Moreover, high baseline S100B in serum was associated with a greater response to antidepressant treatment [72]. These findings suggest that S100B could also be a potential peripheral biomarker for stress-related mood disorders or treatment response [68,73]. However, this statement has to be taken with caution, as both increased and decreased levels were reported in MDD.

It is important to mention that other levels of glial markers were reported to be altered in the periphery. For example, glial-derived neurotrophic factor (GDNF) is reduced in the blood samples of remitted patients with MDD compared to healthy controls [74]. Interestingly, GDNF is mainly synthesized and secreted from neurons in a healthy brain, but during an insult or immune response, its de novo synthesis occurs in glial cells [75]. GDNF is typically upregulated in response to CNS damage to help mediate the growth, differentiation, or survival of neurons [76]. Therefore, one can speculate that a reduction of GDNF indicates a lack of neuroprotective capabilities in the astrocytes of patients with MDD, but this hypothesis must be examined.

Several genes and proteins that are key to astroglial function were reported to be altered in the brains of MDD patients. For example, reductions of aquaporin 4 (AQP4), the predominant water channel of the CNS involved in the coverage of blood vessels by astrocytic endfeet, were described in the orbitofrontal cortex from MDD patients [62]. Another example, MDD post-mortem studies have consistently reported reductions in gene and protein expression of Cx43 and Cx30 (two astrocytic gap-junction proteins) in the dorsolateral PFC, orbitofrontal cortex, and locus coeruleus [58,77,78] (Figure 2). As astrocytes communicate with one another via gap junctions and hemichannels that are permeable to small molecules, including calcium (Ca^2+^), inositol 1,4,5-trisphosphate, sodium, potassium, and reactive oxygen species [79,80], reductions of these proteins can be detrimental to astroglial signaling and neuronal function [81,82].

Another key astrocytic protein change described in MDD brains is the reduction of PFC glutamate transporters, such as the excitatory amino-acid transporter 2 (EAAT2) [57,58] (Figure 1). Indeed, one of the most important roles astrocytes play at the synapse is the reuptake and recycling of neurotransmitters at the synaptic cleft [3,83,84]. Astrocytes are primarily responsible for the reuptake of the excitatory neurotransmitter glutamate via the EAAT1 (also known as glutamate aspartate transporter (GLAST) in mice) and EAAT2 (also known as glutamate transport 1 (GLT1) in mice) [85]. EAAT2/GLT1 transport accounts for over 90% of the reuptake of glutamate from the synaptic cleft to prevent glutamate-induced excitotoxicity [86,87] and for the recycling of glutamate via astrocyte-specific glutamine synthetase [86,87]. Reduced mRNA and protein expression of astrocyte-specific glutamine synthetase (GS), EAAT1, and EAAT2 has been observed in MDD post-mortem studies [57,58,88] (Figure 2). In line with these findings, studies have also reported a significant increase in glutamate levels in the PFC of MDD patients [89,90,91,92] (Figure 2). These changes are associated with decreased glutamatergic (NMDA and AMPA) receptor subunits, their anchor protein PSD95 [93], and metabotropic receptors in PFC, cingulate cortex, thalamus, hippocampus, and other cortical regions [94]. These data suggest a contribution of the observed astroglia pathology in the glutamatergic dysfunction associated with MDD, which is still not fully understood.

Astrocytes also regulate the activity of gamma-aminobutyric acid (GABA)-ergic neurons, since glutamine, the precursor of GABA, is provided by astrocytes via the glutamate–glutamine cycle [86,95]. In GABAergic neurons, glutamine is converted into GABA in the presence of glutamic acid decarboxylase and then released into the synaptic cleft. Astrocytes also reuptake the excess GABA from the synaptic cleft via the sodium- and chloride-dependent GABA transporter 1/3 (GAT1/3) [85]. After reuptake, GABA is reconverted into glutamate via the tricarboxylic acid (TCA) cycle and is further transported to GABAergic neurons for GABA synthesis (referred to as the glutamine-GABA cycle) [96]. Astrocytes can also synthesize GABA via putrescine, which is initiated by either diamine oxidase or monoamine oxidase-B (MAO-B) [95]. Interestingly, GABAergic dysfunctions have been reported in MDD patients with decreased GABA levels in the plasma, CSF, and cerebral cortex, and changes in the expression of GABAA receptors subunit genes were reported [97,98,99,100,101,102,103,104]. Altogether, these observations suggest that astroglial dysfunctions could contribute to the GABAergic deficits associated with MDD. However, the potential relationship between these changes and the link to the expression of the symptoms remains unclear; thus, this is an important point for further study.

Finally, numerous studies have described the glucose metabolic dysfunction in depressed patients being centralized in the PFC and hippocampus [105,106,107,108,109]. Considering astroglia’s unique anatomical location, the ability to take up glucose directly from capillaries [105,106], and the greater glucose utilization compared to neurons [107], it was suggested that astroglial functional impairment may contribute to the glucose metabolism changes observed using positron emission tomography (PET) imaging [108] and to the described reductions associated with MDD found using this approach [109,110,111]. Together, this suggests that the astroglial dysfunction would be detrimental to glutamatergic, GABAergic neurons, and, most probably, all neural cell populations, therefore playing a major role in the brain activity changes associated with MDD.

### 3.2. Evidence of Astroglial Dysfunction in PTSD

As aforementioned, astrocyte dysfunction has also been speculated to play a role in PTSD pathology, but the clinical literature is sparse, mostly because of limited access to the brains of patients with PTSD for post-mortem investigation. This hypothesis is primarily based on rodent model findings; however, indirect evidence exists. Indeed, a recent PET-imaging study showed that the reduction binding of a ligand selective of the enzyme MAO-B in patients with PTSD and attributed these changes to reduced astroglial density [112]. Indeed, recent works suggest that MAOA-B ligand binding could be an in vivo biomarker of astroglial density. This is based on the fact that MAO-B is a monoamine-metabolizing enzyme located primarily in astrocytes (and in monoaminergic neurons) and that increased MAOA-B ligand binding was reported in conditions with increased astrogliosis [112]. Interestingly the reductions in MAO-B ligand binging that were found were greater in PTSD patients with comorbid depression [112]. Genome-wide association studies (GWAS) have also noted alterations in the levels of several genes preferentially expressed in glutamatergic, GABAergic neurons, and astrocytes associated with PTSD [113]. Similarly, a recent single-nucleus RNA-sequencing study, investigating the cell-type-specific transcriptomic alterations in dorsolateral PFC astrocytes and excitatory and inhibitory neurons, identified differentially regulated genes in astroglia that were specific to PTSD compared to MDD samples [114]. Astrocyte dysfunction in PTSD is still an emerging area of investigation. Comparative post-mortem brain studies specifically examining astroglial dysfunction should be conducted to validate these findings and investigate them further. This is particularly important given the ample evidence discussed in the next section regarding the astroglial dysfunctions found in animal models of stress that are relevant to both MDD and PTSD research.

## 4. Astroglial Changes in Animal Models of Stress

Chronic stress exposure has often been used in rodents to model cellular and behavioral features relevant to MDD pathology and symptomology. Models of stress in rodents can include genetic models, surgical models such as bulbectomy, chronic restraint stress (CRS), social stress such as social defeat, and chronic variable stress (CVS) such as unpredictable chronic mild stress (UCMS) [115,116,117]. In these models, chronic stress induces cellular alterations in neuronal and non-neuronal cell populations that are similar to those observed in MDD [19,98,118,119]. Regarding astroglia, chronic stress was shown to reduce PFC GFAP expression or GFAP+ astrocyte number [118,119,120,121,122] and astrocyte morphology complexity [123]. Similar reductions in GFAP expression or GFAP cell number were reported in the hippocampus [119,120,124,125,126,127,128,129], as well as GFAP cell atrophy [130,131]. Few studies, however, found no changes or increased GFAP in this brain region [121,132]. A recent study investigating astrocytes at the network level confirmed astroglial atrophy following chronic stress exposure and demonstrated an impairment of the strength of astrocyte syncytial coupling within the hippocampus and PFC [133]. Chronic stress in rodents also causes astroglial changes in other brain regions, such as the amygdala, but the data are inconsistent [119,120,130,131,133]. Similar inconsistent results were reported with S100B+ cell density or expression [133,134].

Animals exposed to chronic stress also show changes in the expression of the key astroglial genes associated with astroglial plasticity and synaptic formation, as well as decreased growth-factor expression and the suppression of associated signaling pathways [135]. Many protein dysregulations in rodent chronic stress models parallel those seen in MDD brains. GLT1/EAAT2 has especially been found to be downregulated in the periaqueductal gray matter [136], PFC [137,138], and hippocampus [139,140], but some studies reported no changes or increased expression level following chronic stress [141,142,143]. Additional changes include a reduction of glucocorticoid receptor (GR) expression in astrocytes [32] and significant decreases in Cx43 gap-junction function and expression in the PFC [144,145], as summarized in [27]. Others have also reported decreased gliotransmitter release, such as reduced astrocyte-derived ATP in mice subjected to chronic social defeat [146] or increased tonic GABA inhibition in the PFCs of rats subjected to stress, which have been attributed to the regulation of astrocytic release of GABA [147].

Similar changes were reported in rodent models relevant to PTSD, which include inescapable electric foot shock, aversive sensory stimulation (predator odor), and prolonged single-stress exposure [148]. In these models, reduced GFAP or GLT1 expression was described in the hippocampus [149,150,151,152]. Decreased GFAP+ astroglial density in the PFC [149] and hippocampus [152], as well as GFAP+ astrocyte cell-body atrophy and process thinning also being reported [153,154]. It should be noted that some studies report increased GFAP astrocytes following social isolation [153] or footshock exposure [152,155], but the inconsistency in the reported findings may be attributed to the type of acute stress model used or the interval between the stress exposure and the astroglial measurement.

## 5. Effect of Astrocyte Manipulations on Emotion-Related Behavior

**Figure 3:**
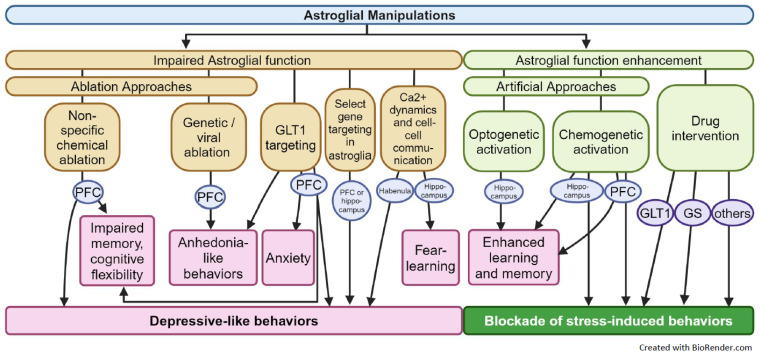
Schematic representation of the key findings related to the behavioral effects of experimental astroglial manipulations, with a special focus on emotion-related behaviors relevant to MDD and PTSD pathologies. Abbreviations: GLT1: glial glutamate transporter 1, GS: glutamine synthetase, PFC: prefrontal cortex.

Rodent experimentation has been instrumental in testing the “direct” or “causal” involvement of astroglia and astroglial dysfunction in the development of anxiety- and depressive-like behavior. Indeed, attempts were made to mimic the behavioral effects of chronic stress models by manipulating astrocytes or the expression of key astroglial proteins shown to be affected by stress (Figure 3). The first studies that investigated the specific role of astroglia on behavior used cell-ablation approaches through pharmacological means to determine if there was a causal relationship between astrocyte reductions and emotion- or cognition-related behaviors [118,156]. Indeed, infusion of non-specific gliotoxins in the PFC was reported to induce depressive-like deficits [118] and impair cognitive flexibility [156]. This was confirmed in several studies [143,157,158,159,160,161]. More recently, we showed that selective GFAP cell loss in the PFC induces anhedonia-like behavior [162]. It was also shown that intracerebral infusion of the GLT-1 inhibitor, dihydrokainic acid (DHK), induces anhedonia-like behaviors [163] and cognitive impairment [164]. PFC infusion of another inhibitor targeting both GLAST and GLT1 was shown to induce anxiety-like behavior [165]. These results were confirmed by studies showing that GLAST and GLT1 knockdowns in the PFC induced depressive-like behavior [166]. It is important to mention that some studies have demonstrated the upregulation of GLT1 in stress conditions [167,168,169] and that an acute pharmacological blockade of GLT1 may have antidepressant-like effects [168,170], but again, the variability in the stress paradigm used, length of the stress exposure, or kinetics of pharmacological interventions may be factors in the variable observations.

The involvement of astroglial regulation of emotion-related behaviors was also demonstrated through astroglia-specific gene or protein manipulations. To cite a few examples, GR knockout in the GFAP+ astrocytes of the PFC was shown to be sufficient to induce depressive-like behaviors, and restoring GR expression prevented these deficits [32]. The astroglial potassium channel (Kir4.1, an ATP-sensitive inward rectifier potassium channel 10) in the lateral habenula was also reported to regulate depressive-like behaviors [171]. Overexpression of Cx43 in the hippocampus prevented depressive-like behaviors and spatial memory deficits induced by maternal separation stress [172]. Together, these findings support the idea that astroglial dysfunctions in various brain regions may contribute to the development of emotion-related behaviors and cognitive impairment associated with stress.

With recent advances in cell- and region-specific viral and genetic targeting or cell activity monitoring, the investigation of the consequences of astroglial-specific manipulations and/or the effects of specific interventions on astrocyte activity has yielded new insights into the role of astrocytes in the modulation of emotion-related behavior. Most of these studies capitalized on the fact that astroglial activity changes rely on fluctuations in intercellular Ca^2+^ concentrations [173,174]. Using Ca^2+^ imaging in vivo, it was shown that the activity of hippocampal astrocytes is increased in anxiogenic conditions and that optogenetic activation of these cells is sufficient to induce anxiety [175]. Oppositely, hippocampal GFAP+ astroglial activation was sufficient to reduce stress-enhanced fear learning [176]. In a similar context, specific knockdown of the Ca^2+^ channel Orai1 in astrocytes, which downregulated genes in inflammation and metabolism, as well as reduced cellular metabolites and ATP production, was shown to blunt lipopolysaccharide-induced depressive-like behaviors, such as anhedonia and learned helplessness [177]. It was also found that mice deficient in inositol triphosphate type 2 receptors (IP3R2), a receptor known to diminish Ca^2+^ fluctuations in astroglia, have disrupted astrocytic excitability, impaired gliotransmitter release, and altered startle response [178]; however, IP3R2 conditional knockout mice display no major anxiety or cognitive deficits [179]. Several other studies have investigated the involvement of changes in astroglial activity on memory or fear learning. Indeed, Delacorte et al. [180] demonstrated that L-alpha-amino-adipic acid (gliotoxin)-induced glial loss and, inversely, chemogenetic modulation of PFC GFAP+ astrocytes, using a designer receptor exclusively activated by designer drugs (DREADD) approach, were able to alter recognition-memory performance in a novel object-recognition task (Figure 3). Similarly, DREADD-mediated activation of astrocytes in the CA1 region of the hippocampus increases neuronal activity, triggers the induction of long-term potentiation (LTP), and improves performance in T-maze and hippocampal-dependent memory tests, such as contextual fear learning [181,182]. Oppositely, it was shown that the activation of the Gi pathway in CA1 hippocampal astrocytes impaired retrieval in contextual fear-learning tasks and that this was due to deficient astrocytic modulation of the circuit connecting CA1 neurons to ACC neurons [183]. Consistent with this, enhanced LTP in synapses and improved memory performance were observed following optogenetic activation of the CA1 hippocampal astrocytes using melanopsin [184]. Interestingly, a recent study using a fiber photometry Ca^2+^ imaging technique to measure neuronal and astroglial activity simultaneously and similar CA1 optogenetic manipulations involved neuronal–astrocytic coupling as a shared mechanism, enabling both natural and artificially induced memory retrieval and the behavioral expression of fear [185]. The same group also showed that basolateral amygdala astrocytes robustly responded to footshock during contextual fear acquisition, and their activity remained elevated in comparison to control animals. But, they found no change in the astrocytic activity and/or freezing behavior following chemogenetic inhibition of the basolateral amygdala neurons [186]. Other recent work using chemogenetic activation to increase GFAP+ astroglia activity in the amygdala showed that astroglial activation reduced fear-related anxiety behavior through the mechanisms of disrupted fear-memory consolidation and decreased contextual memory [187]. Altogether, these studies highlight the important role of astroglia in anxiety and fear-memory regulation, key dimensions of impaired PTSD.

Most of the aforementioned studies focused on acute or short-term manipulations of astroglial activity, but a few have investigated the effects of chronic activation in chronic stress or chronic stress-like conditions. Indeed, in a recent study, our lab recently demonstrated that anhedonia-like deficits were observed following selective ablation of PFC GFAP+ astrocytes using a diphtheria-toxin (DT) receptor-expressing virus and DT injection [162] (Figure 3). Importantly, the selective PFC GFAP+ cell depletion induced anhedonia-like deficits within 2–3 days, which is a behavior usually observed following weeks of chronic stress exposure. Conversely, chronic enhancement of GFAP+ astroglia activity in the PFC reversed chronic stress-induced anhedonia-like deficits [162]. Note that, in this study, we showed no effect of the GFAP cell ablation or activation on anxiety-like behaviors, which may oppose the findings previously discussed, where optogenetics or acute chemogenetics were employed. A concomitant recent study by González-Arias et al. [188] investigated PFC GFAP+ cell activity in juvenile mice subjected to chronic corticosterone treatment and showed impairments of astroglial activity during tests measuring anxiety or social interaction deficits that were reversed in mice by chronic chemogenetic enhancement of GFAP+ cell activity. Another study used chronic chemogenetic activation to investigate astroglial modulation of alcohol consumption [189]. This is relevant considering the greater glial loss reported in MDD patients with co-morbid alcohol-use disorder compared to the MDD-only cohort [61] and the bidirectional link between MDD and alcoholism [190,191]. In this context, it was shown that PFC astroglial ablation, impairment of astroglial communication through gap-junction [192], or modulation of astroglial activity [189] was associated with increased ethanol consumption and intoxication effects. Altogether, these studies involve chronic astroglial dysfunctions in anhedonia, anxiety, and alcohol consumption, all behavioral features strongly associated with chronic stress and MDD.

Because of the limited tools and approaches to manipulate astroglia in adult animals, in particular chronically, most of the studies have used the DREADD approach or single-gene manipulations using viruses focused on GFAP+ cell populations. As they employ the GFAP promotor for cell targeting, further research should also investigate the potential implication of other astroglial subpopulations. Nevertheless, these studies showed that the changes observed following artificial impairment of GFAP+ cell function are reminiscent of the changes induced by chronic stress, and oppositely, enhancing GFAP+ cell function can prevent or reverse the effects of stress. This demonstrates GFAP+ astrocytes in different brain regions, and the resulting changes occurring in the neighboring neurons and synapses can regulate emotion- and cognition-related behaviors. However, the specific consequences of these astroglial changes on the surrounding neuronal function and how these changes affect behavior remain unclear. Future studies should focus on identifying the neuronal consequences of astroglial dysfunction, as this line of investigation may yield new neuronal and astroglial targets for antidepressant development.

## 6. Targeting Astrocytes for Treatment

The idea of targeting astroglia for antidepressant development has grown over the years [18,193,194,195,196,197]. However, it is important to first mention that enhanced astroglial function is observed following treatment with classical antidepressants, such as selective serotonin reuptake inhibitors (SSRIs) and tricyclic antidepressants (TCAs), and may, in part, contribute to their action. This is not surprising, as astroglia express several serotonergic receptors and MAOs [198] that, when activated trigger cellular cascades known to be involved in mood regulation. For example, brain-derived neurotrophic factor (BDNF) is a neurotrophic factor shown to be key in the behavioral and neuroplastic action of classical or rapid-acting antidepressant treatments, such as ketamine [199,200,201]. BDNF is also released by the astroglia, and several in vitro studies reported astroglial release of BDNF following SSRI or TCA exposure [202,203,204]. In addition, SSRIs increase astroglial glucose uptake and metabolism [202] Cx43 expression [205] and ATP gliotransmission [206], mechanisms that were shown to alter emotion-related behaviors.

There is also more direct evidence that some classical antidepressants may partially depend on enhancing astroglial function to exert their action. Indeed, it was shown that the behavioral antidepressant effects of fluoxetine in animals subjected to chronic stress are blunted in aquaporin-4 knockout mice [207]. Similarly, gap-junction blockade in the PFC, which can induce anhedonia- and anxiety-like behavior in animals [145], was shown to prevent fluoxetine antidepressant action [208]. In addition, GLT1 or GLAST knockdowns in the PFC have been shown to induce depressive-like deficits [209]. These transporters were implicated in the antidepressant action of ketamine [210,211,212,213], deep brain stimulation [140,157], fluoxetine [140], and others [160]. The fact that some antidepressant treatments require key astroglial proteins or functions to exert their behavioral effects suggests that some antidepressants may prevent astroglial loss/dysfunction induced by chronic stress. This is supported by data collected from rodents, which show blunted chronic stress-induced astroglial density reduction following fluoxetine treatment [124], and from humans, where no significant decrease in astroglial density was found in treated MDD patients [52]. However, this may need to be systematically tested for a few antidepressants of each class, as it was shown that fluoxetine treatment can reverse the chronic stress-induced GFAP reductions [124], but not citalopram [126]. Interestingly, recent work suggested that the fast-acting properties of some antidepressants may coincide with the reversal of chronic stress effects on the astroglial density or function [214] or rely on astroglia for their long-lasting effects [214,215]

As evidence of the beneficial effects of enhancing key astroglial functions on emotion-related effects is growing, interest in targeting astroglia for treatment is rising. Less advanced avenues of investigation for antidepressant development include drugs that promote endogenous ATP release from astrocytes [146] or glutamine supplementation, which can reverse stress-induced depressive-like behaviors [137]. One line of research showing promise for antidepressant development relies on targeting astroglial glutamate transport. Indeed, several drugs can increase GLT1 (or GLAST) expression or activity and have antidepressant-like effects in rodents, including ceftriaxone [a beta-lactam antibiotic] [216], riluzole [a Na+ channel blocker] [217,218], guanosine [a guanine-based purine] [219,220], and harmine [a beta-carboline alkaloid] [221,222], further supporting astroglial GLT1 as a potential antidepressant target. However, it is important to mention that the requirement of astroglia or GLT1 for the antidepressant activity of these drugs was not directly tested using, for example, KO or viral approaches. These drugs are not specific to GLT1 and may exert their antidepressant actions through other (or additional) cellular mechanisms, e.g., by increasing BDNF and its pathways (riluzole, ceftriaxone, and harmine) [222,223,224], inducing neuro/synapogenesis [131,225], GABA modulation (harmine) [226,227] or the regulation of Akt, NFKB, or mTOR signaling cascades (ceftriaxone, guanosine) [228,229]. In addition, interest in some of these non-specific drugs as antidepressants, in particular, riluzole, is further mitigated by a double-blind placebo-controlled study showing no benefit of riluzole augmentation in MDD patients [230,231], which opposed several open-label studies reporting potential antidepressant effects for riluzole [232,233]. Similar negative results were reported in a PTSD trial, albeit some beneficial effects were found on hyperarousal [234]. Guanosine, harmine, and ceftriaxone have not yet been tested in clinical settings in patients with MDD or PTSD, but rodent research supports potential antidepressant action in rodent models, which is relevant for both disorders [219,221,235]. Importantly, while riluzole, ceftriaxone, and harmine were shown to require chronic administration for antidepressant action, guanosine has rapid-acting antidepressant-like properties similar to ketamine and was shown to potentiate ketamine’s effects on GLT1 and GS activity [229,236]. Again, the requirement of GLT1/astroglia for the guanosine antidepressant action needs to be demonstrated, as the rapid-acting antidepressant effects of ketamine were shown to be independent of GLT1 [215]. Altogether, these findings support targeting GLT1 as a viable avenue for the treatment of MDD and PTSD, but the development of selective compounds is crucially needed [237]. Such drugs would be relevant for the treatment of many illnesses, as glutamate spillover is a common feature of several mood disorders, addiction, and neurodegenerative diseases [91,92].

## 7. Conclusions

In this review, we offered a snapshot of the current literature outlining the connections between astroglial deficits in mood disorders and stress in both preclinical and clinical research. We also detailed major findings supporting astroglia as a key partner in the regulation of emotion-related behaviors and highlighted future areas of research for investigating novel antidepressants that target astrocytes. With the recent progress in adapting optical, genetic, and biochemical techniques to investigate astroglia, we predict that future research will yield a better understanding of the molecular mechanisms underlying astroglial dysfunctions and their contribution to the maladaptive neuronal and synaptic changes associated with mood disorders, as these are necessary steps to devise ways to target astroglia for the treatment of stress-related illnesses.

## References

[1] Saint-Martin M., Goda Y. (2022). Astrocyte-Synapse Interactions and Cell Adhesion Molecules. FEBS J..

[2] Verkhratsky A., Nedergaard M. (2018). Physiology of Astroglia. Physiol. Rev..

[3] Hussaini S.M.Q., Jang M.H. (2018). New Roles for Old Glue: Astrocyte Function in Synaptic Plasticity and Neurological Disorders. Int. Neurourol. J..

[4] Oberheim N.A., Goldman S.A., Nedergaard M. (2012). Heterogeneity of Astrocytic Form and Function. Methods Mol. Biol..

[5] Endo F., Kasai A., Soto J.S., Yu X., Qu Z., Hashimoto H., Gradinaru V., Kawaguchi R., Khakh B.S. (2022). Molecular Basis of Astrocyte Diversity and Morphology across the CNS in Health and Disease. Science.

[6] Qian Z., Qin J., Lai Y., Zhang C., Zhang X. (2023). Large-Scale Integration of Single-Cell RNA-Seq Data Reveals Astrocyte Diversity and Transcriptomic Modules across Six Central Nervous System Disorders. Biomolecules.

[7] Westergard T., Rothstein J.D. (2020). Astrocyte Diversity: Current Insights and Future Directions. Neurochem. Res..

[8] Bugiani M., Plug B.C., Man J.H.K., Breur M., van der Knaap M.S. (2022). Heterogeneity of White Matter Astrocytes in the Human Brain. Acta Neuropathol..

[9] Köhler S., Winkler U., Hirrlinger J. (2021). Heterogeneity of Astrocytes in Grey and White Matter. Neurochem. Res..

[10] Sofroniew M.V., Vinters H.V. (2010). Astrocytes: Biology and Pathology. Acta Neuropathol..

[11] Du J., Yi M., Zhou F., He W., Yang A., Qiu M., Huang H. (2021). S100B Is Selectively Expressed by Gray Matter Protoplasmic Astrocytes and Myelinating Oligodendrocytes in the Developing CNS. Mol. Brain.

[12] Yang Z., Wang K.K. (2015). Glial Fibrillary Acidic Protein: From Intermediate Filament Assembly and Gliosis to Neurobiomarker. Trends Neurosci..

[13] Werkman I.L., Dubbelaar M.L., van der Vlies P., de Boer-Bergsma J.J., Eggen B.J.L., Baron W. (2020). Transcriptional Heterogeneity between Primary Adult Grey and White Matter Astrocytes Underlie Differences in Modulation of In Vitro Myelination. J. Neuroinflammation.

[14] Escartin C., Galea E., Lakatos A., O’Callaghan J.P., Petzold G.C., Serrano-Pozo A., Steinhäuser C., Volterra A., Carmignoto G., Agarwal A. (2021). Reactive Astrocyte Nomenclature, Definitions, and Future Directions. Nat. Neurosci..

[15] Sofroniew M.V. (2020). Astrocyte Reactivity: Subtypes, States, and Functions in CNS Innate Immunity. Trends Immunol..

[16] Fan Y.Y., Huo J. (2021). A1/A2 Astrocytes in Central Nervous System Injuries and Diseases: Angels or Devils?. Neurochem. Int..

[17] El Fatimi H., Khalki L. (2023). Involvement of Glial Cells in the Pathophysiology and Treatment of Depression. Physiology and Function of Glial Cells in Health and Disease.

[18] Sanacora G., Banasr M. (2013). From Pathophysiology to Novel Antidepressant Drugs: Glial Contributions to the Pathology and Treatment of Mood Disorders. Biol. Psychiatry.

[19] Banasr M., Sanacora G., Esterlis I. (2021). Macro- and Microscale Stress–Associated Alterations in Brain Structure: Translational Link with Depression. Biol. Psychiatry.

[20] Li B., Zhang D., Verkhratsky A. (2022). Astrocytes in Post-Traumatic Stress Disorder. Neurosci. Bull..

[21] Zhou X., Xiao Q., Xie L., Yang F., Wang L., Tu J. (2019). Astrocyte, a Promising Target for Mood Disorder Interventions. Front. Mol. Neurosci..

[22] Zhou B., Zuo Y.X., Jiang R.T. (2019). Astrocyte Morphology: Diversity, Plasticity, and Role in Neurological Diseases. CNS Neurosci. Ther..

[23] McEwen B.S., Akil H. (2020). Revisiting the Stress Concept: Implications for Affective Disorders. J. Neurosci..

[24] Juster R.P., McEwen B.S., Lupien S.J. (2010). Allostatic Load Biomarkers of Chronic Stress and Impact on Health and Cognition. Neurosci. Biobehav. Rev..

[25] Matos T.M., Souza-Talarico J.N. (2019). How Stress Mediators Can Cumulatively Contribute to Alzheimer’s Disease an Allostatic Load Approach. Dement. Neuropsychol..

[26] McEwen B.S. (2017). Neurobiological and Systemic Effects of Chronic Stress. Chronic Stress.

[27] Miguel-Hidalgo J.J. (2022). Astroglia in the Vulnerability to and Maintenance of Stress-Mediated Neuropathology and Depression. Front. Cell Neurosci..

[28] Murphy-Royal C., Johnston A.D., Boyce A.K.J., Diaz-Castro B., Institoris A., Peringod G., Zhang O., Stout R.F., Spray D.C., Thompson R.J. (2020). Stress Gates an Astrocytic Energy Reservoir to Impair Synaptic Plasticity. Nat. Commun..

[29] Silverman M.N., Sternberg E.M. (2012). Glucocorticoid Regulation of Inflammation and Its Functional Correlates: From HPA Axis to Glucocorticoid Receptor Dysfunction. Ann. N. Y. Acad. Sci..

[30] Smith S.M., Vale W.W. (2006). The Role of the Hypothalamic-Pituitary-Adrenal Axis in Neuroendocrine Responses to Stress. Dialogues Clin. Neurosci..

[31] Tertil M., Skupio U., Barut J., Dubovyk V., Wawrzczak-Bargiela A., Soltys Z., Golda S., Kudla L., Wiktorowska L., Szklarczyk K. (2018). Glucocorticoid Receptor Signaling in Astrocytes Is Required for Aversive Memory Formation. Transl. Psychiatry.

[32] Lu C.L., Ren J., Mo J.W., Fan J., Guo F., Chen L.Y., Wen Y.L., Li S.J., Fang Y.Y., Wu Z.F. (2022). Glucocorticoid Receptor-Dependent Astrocytes Mediate Stress Vulnerability. Biol. Psychiatry.

[33] Murphy-Royal C., Gordon G.R., Bains J.S. (2019). Stress-Induced Structural and Functional Modifications of Astrocytes-Further Implicating Glia in the Central Response to Stress. Glia.

[34] Luarte A., Cisternas P., Caviedes A., Batiz L.F., Lafourcade C., Wyneken U., Henzi R. (2017). Astrocytes at the Hub of the Stress Response: Potential Modulation of Neurogenesis by MiRNAs in Astrocyte-Derived Exosomes. Stem Cells Int..

[35] Olejniczak M., Kotowska-Zimmer A., Krzyzosiak W. (2018). Stress-Induced Changes in MiRNA Biogenesis and Functioning. Cell Mol. Life Sci..

[36] Ding R., Su D., Zhao Q., Wang Y., Wang J.Y., Lv S., Ji X. (2023). The Role of MicroRNAs in Depression. Front. Pharmacol..

[37] Luarte A., Henzi R., Fernández A., Gaete D., Cisternas P., Pizarro M., Batiz L.F., Villalobos I., Masalleras M., Vergara R. (2020). Astrocyte-Derived Small Extracellular Vesicles Regulate Dendritic Complexity through MiR-26a-5p Activity. Cells.

[38] Du L., Jiang Y., Sun Y. (2021). Astrocyte-Derived Exosomes Carry MicroRNA-17-5p to Protect Neonatal Rats from Hypoxic-Ischemic Brain Damage via Inhibiting BNIP-2 Expression. Neurotoxicology.

[39] Leggio L., L’Episcopo F., Magrì A., Ulloa-Navas M.J., Paternò G., Vivarelli S., Bastos C.A.P., Tirolo C., Testa N., Caniglia S. (2022). Small Extracellular Vesicles Secreted by Nigrostriatal Astrocytes Rescue Cell Death and Preserve Mitochondrial Function in Parkinson’s Disease. Adv. Healthc. Mater..

[40] Ranjit S., Patters B.J., Gerth K.A., Haque S., Choudhary S., Kumar S. (2018). Potential Neuroprotective Role of Astroglial Exosomes against Smoking-Induced Oxidative Stress and HIV-1 Replication in the Central Nervous System. Expert. Opin. Ther. Targets.

[41] Weiss Roberts L. (2019). Textbook of Psychiatry.

[42] Wu Z., Fang Y. (2014). Comorbidity of Depressive and Anxiety Disorders: Challenges in Diagnosis and Assessment. Shanghai Arch. Psychiatry.

[43] Hirschfeld R.M. (2001). The Comorbidity of Major Depression and Anxiety Disorders: Recognition and Management in Primary Care. Prim. Care Companion J. Clin. Psychiatry.

[44] Groen R.N., Ryan O., Wigman J.T.W., Riese H., Penninx B.W.J.H., Giltay E.J., Wichers M., Hartman C.A. (2020). Comorbidity between Depression and Anxiety: Assessing the Role of Bridge Mental States in Dynamic Psychological Networks. BMC Med..

[45] Lamers F., van Oppen P., Comijs H.C., Smit J.H., Spinhoven P., van Balkom A.J., Nolen W.A., Zitman F.G., Beekman A.T., Penninx B.W. (2011). Comorbidity Patterns of Anxiety and Depressive Disorders in a Large Cohort Study: The Netherlands Study of Depression and Anxiety (NESDA). J. Clin. Psychiatry.

[46] Rajkowska G., Stockmeier C.A. (2013). Astrocyte Pathology in Major Depressive Disorder: Insights from Human Postmortem Brain Tissue. Curr. Drug Targets.

[47] Cotter D.R., Pariante C.M., Everall I.P. (2001). Glial Cell Abnormalities in Major Psychiatric Disorders: The Evidence and Implications. Brain Res. Bull..

[48] Cotter D., Mackay D., Chana G., Beasley C., Landau S., Everall I.P. (2002). Reduced Neuronal Size and Glial Cell Density in Area 9 of the Dorsolateral Prefrontal Cortex in Subjects with Major Depressive Disorder. Cereb. Cortex.

[49] Ongür D., Drevets W.C., Price J.L. (1998). Glial Reduction in the Subgenual Prefrontal Cortex in Mood Disorders. Proc. Natl. Acad. Sci. USA.

[50] Rajkowska G., Miguel-Hidalgo J.J., Wei J., Dilley G., Pittman S.D., Meltzer H.Y., Overholser J.C., Roth B.L., Stockmeier C.A. (1999). Morphometric Evidence for Neuronal and Glial Prefrontal Cell Pathology in Major Depression. Biol. Psychiatry.

[51] Gittins R.A., Harrison P.J. (2011). A Morphometric Study of Glia and Neurons in the Anterior Cingulate Cortex in Mood Disorder. J. Affect. Disord..

[52] Cobb J.A., O’Neill K., Milner J., Mahajan G.J., Lawrence T.J., May W.L., Miguel-Hidalgo J., Rajkowska G., Stockmeier C.A. (2016). Density of GFAP-Immunoreactive Astrocytes Is Decreased in Left Hippocampi in Major Depressive Disorder. Neuroscience.

[53] O’Leary L.A., Belliveau C., Davoli M.A., Ma J.C., Tanti A., Turecki G., Mechawar N. (2021). Widespread Decrease of Cerebral Vimentin-Immunoreactive Astrocytes in Depressed Suicides. Front. Psychiatry.

[54] Khundakar A., Morris C., Oakley A., Thomas A.J. (2011). A Morphometric Examination of Neuronal and Glial Cell Pathology in the Orbitofrontal Cortex in Late-Life Depression. Int. Psychogeriatr..

[55] Khundakar A.A., Morris C.M., Oakley A.E., Thomas A.J. (2011). Cellular Pathology within the Anterior Cingulate Cortex of Patients with Late-Life Depression: A Morphometric Study. Psychiatry Res. Neuroimaging.

[56] Cobb J.A., Simpson J., Mahajan G.J., Overholser J.C., Jurjus G.J., Dieter L., Herbst N., May W., Rajkowska G., Stockmeier C.A. (2013). Hippocampal Volume and Total Cell Numbers in Major Depressive Disorder. J. Psychiatr. Res..

[57] Miguel-Hidalgo J.J., Waltzer R., Whittom A.A., Austin M.C., Rajkowska G., Stockmeier C.A. (2010). Glial and Glutamatergic Markers in Depression, Alcoholism, and Their Comorbidity. J. Affect. Disord..

[58] Nagy C., Suderman M., Yang J., Szyf M., Mechawar N., Ernst C., Turecki G. (2015). Astrocytic Abnormalities and Global DNA Methylation Patterns in Depression and Suicide. Mol. Psychiatry.

[59] Si X., Miguel-Hidalgo J.J., O’Dwyer G., Stockmeier C.A., Rajkowska G. (2004). Age-Dependent Reductions in the Level of Glial Fibrillary Acidic Protein in the Prefrontal Cortex in Major Depression. Neuropsychopharmacology.

[60] Rajkowska G., Legutko B., Moulana M., Syed M., Romero D.G., Stockmeier C.A., Miguel-Hidalgo J.J. (2018). Astrocyte Pathology in the Ventral Prefrontal White Matter in Depression. J. Psychiatr. Res..

[61] Miguel-Hidalgo J.J., Wei J., Andrew M., Overholser J.C., Jurjus G., Stockmeier C.A., Rajkowska G. (2002). Glia Pathology in the Prefrontal Cortex in Alcohol Dependence with and without Depressive Symptoms. Biol. Psychiatry.

[62] Rajkowska G., Hughes J., Stockmeier C.A., Javier Miguel-Hidalgo J., Maciag D. (2013). Coverage of Blood Vessels by Astrocytic Endfeet Is Reduced in Major Depressive Disorder. Biol. Psychiatry.

[63] Michel M., Fiebich B.L., Kuzior H., Meixensberger S., Berger B., Maier S., Nickel K., Runge K., Denzel D., Pankratz B. (2021). Increased GFAP Concentrations in the Cerebrospinal Fluid of Patients with Unipolar Depression. Transl. Psychiatry.

[64] Klempan T.A., Sequeira A., Canetti L., Lalovic A., Ernst C., ffrench-Mullen J., Turecki G. (2009). Altered Expression of Genes Involved in ATP Biosynthesis and GABAergic Neurotransmission in the Ventral Prefrontal Cortex of Suicides with and without Major Depression. Mol. Psychiatry.

[65] Gos T., Schroeter M.L., Lessel W., Bernstein H.G., Dobrowolny H., Schiltz K., Bogerts B., Steiner J. (2013). S100B-Immunopositive Astrocytes and Oligodendrocytes in the Hippocampus Are Differentially Afflicted in Unipolar and Bipolar Depression: A Postmortem Study. J. Psychiatr. Res..

[66] Schroeter M.L., Abdul-Khaliq H., Diefenbacher A., Blasig I.E. (2002). S100B Is Increased in Mood Disorders and May Be Reduced by Antidepressive Treatment. Neuroreport.

[67] Tural U., Irvin M.K., Iosifescu D. (2022). V Correlation between S100B and Severity of Depression in MDD: A Meta-Analysis. World J. Biol. Psychiatry.

[68] Kozlowski T., Bargiel W., Grabarczyk M., Skibinska M. (2023). Peripheral S100B Protein Levels in Five Major Psychiatric Disorders: A Systematic Review. Brain Sci..

[69] Fang Y., Xiao S.F., Zhang S.Y., Qiu Q., Wang T., Li X. (2016). Increased Plasma S100β Level in Patients with Major Depressive Disorder. CNS Neurosci. Ther..

[70] Rothermundt M., Arolt V., Wiesmann M., Missler U., Peters M., Rudolf S., Kirchner H. (2001). S-100B Is Increased in Melancholic but Not in Non-Melancholic Major Depression. J. Affect. Disord..

[71] Schmidt F.M., Mergl R., Stach B., Jahn I., Schönknecht P. (2015). Elevated Levels of Cerebrospinal Fluid Neuron-Specific Enolase (NSE), but Not S100B in Major Depressive Disorder. World J. Biol. Psychiatry.

[72] Navinés R., Oriolo G., Horrillo I., Cavero M., Aouizerate B., Schaefer M., Capuron L., Meana J.J., Martin-Santos R. (2022). High S100B Levels Predict Antidepressant Response in Patients with Major Depression even When Considering Inflammatory and Metabolic Markers. Int. J. Neuropsychopharmacol..

[73] Levchuk L.A., Roschina O.V., Mikhalitskaya E.V., Epimakhova E.V., Simutkin G.G., Bokhan N.A., Ivanova S.A. (2023). Serum Levels of S100B Protein and Myelin Basic Protein as a Potential Biomarkers of Recurrent Depressive Disorders. J. Pers. Med..

[74] Takebayashi M., Hisaoka K., Nishida A., Tsuchioka M., Miyoshi I., Kozuru T., Hikasa S., Okamoto Y., Shinno H., Morinobu S. (2006). Decreased Levels of Whole Blood Glial Cell Line-Derived Neurotrophic Factor (GDNF) in Remitted Patients with Mood Disorders. Int. J. Neuropsychopharmacol..

[75] Duarte Azevedo M., Sander S., Tenenbaum L. (2020). GDNF, A Neuron-Derived Factor Upregulated in Glial Cells during Disease. J. Clin. Med..

[76] Zhang Z., Sun G.Y., Ding S. (2021). Glial Cell Line-Derived Neurotrophic Factor and Focal Ischemic Stroke. Neurochem. Res..

[77] Ernst C., Nagy C., Kim S., Yang J.P., Deng X., Hellstrom I.C., Choi K.H., Gershenfeld H., Meaney M.J., Turecki G. (2011). Dysfunction of Astrocyte Connexins 30 and 43 in Dorsal Lateral Prefrontal Cortex of Suicide Completers. Biol. Psychiatry.

[78] Miguel-Hidalgo J.J., Wilson B.A., Hussain S., Meshram A., Rajkowska G., Stockmeier C.A. (2014). Reduced Connexin 43 Immunolabeling in the Orbitofrontal Cortex in Alcohol Dependence and Depression. J. Psychiatr. Res..

[79] Theis M., Giaume C. (2012). Connexin-Based Intercellular Communication and Astrocyte Heterogeneity. Brain Res..

[80] Semyanov A., Henneberger C., Agarwal A. (2020). Making Sense of Astrocytic Calcium Signals—from Acquisition to Interpretation. Nat. Rev. Neurosci..

[81] Halassa M.M., Haydon P.G. (2010). Integrated Brain Circuits: Astrocytic Networks Modulate Neuronal Activity and Behavior. Annu. Rev. Physiol..

[82] Turovsky E.A., Braga A., Yu Y., Esteras N., Korsak A., Theparambil S.M., Hadjihambi A., Hosford P.S., Teschemacher A.G., Marina N. (2020). Mechanosensory Signaling in Astrocytes. J. Neurosci..

[83] Newman E.A. (2003). New Roles for Astrocytes: Regulation of Synaptic Transmission. Trends Neurosci..

[84] Shen W., Chen S., Liu Y., Han P., Ma T., Zeng L.H. (2021). Chemogenetic Manipulation of Astrocytic Activity: Is It Possible to Reveal the Roles of Astrocytes?. Biochem. Pharmacol..

[85] Jurga A.M., Paleczna M., Kadluczka J., Kuter K.Z. (2021). Beyond the GFAP-Astrocyte Protein Markers in the Brain. Biomolecules.

[86] Hayashi M.K. (2018). Structure-Function Relationship of Transporters in the Glutamate-Glutamine Cycle of the Central Nervous System. Int. J. Mol. Sci..

[87] Pajarillo E., Rizor A., Lee J., Aschner M., Lee E. (2019). The Role of Astrocytic Glutamate Transporters GLT-1 and GLAST in Neurological Disorders: Potential Targets for Neurotherapeutics. Neuropharmacology.

[88] Choudary P.V., Molnar M., Evans S.J., Tomita H., Li J.Z., Vawter M.P., Myers R.M., Bunney W.E., Akil H., Watson S.J. (2005). Altered Cortical Glutamatergic and GABAergic Signal Transmission with Glial Involvement in Depression. Proc. Natl. Acad. Sci. USA.

[89] Hashimoto K., Sawa A., Iyo M. (2007). Increased Levels of Glutamate in Brains from Patients with Mood Disorders. Biol. Psychiatry.

[90] Haroon E., Miller A.H., Sanacora G. (2017). Inflammation, Glutamate, and Glia: A Trio of Trouble in Mood Disorders. Neuropsychopharmacology.

[91] Popoli M., Yan Z., McEwen B.S., Sanacora G. (2011). The Stressed Synapse: The Impact of Stress and Glucocorticoids on Glutamate Transmission. Nat. Rev. Neurosci..

[92] Sanacora G., Treccani G., Popoli M. (2012). Towards a Glutamate Hypothesis of Depression: An Emerging Frontier of Neuropsychopharmacology for Mood Disorders. Neuropharmacology.

[93] Feyissa A.M., Chandran A., Stockmeier C.A., Karolewicz B. (2009). Reduced Levels of NR2A and NR2B Subunits of NMDA Receptor and PSD-95 in the Prefrontal Cortex in Major Depression. Prog. Neuropsychopharmacol. Biol. Psychiatry.

[94] Esterlis I., DellaGioia N., Pietrzak R.H., Matuskey D., Nabulsi N., Abdallah C.G., Yang J., Pittenger C., Sanacora G., Krystal J.H. (2018). Ketamine-Induced Reduction in MGluR5 Availability Is Associated with an Antidepressant Response: An [11C] ABP688 and PET imaging study in depression. Mol. Psychiatry.

[95] Andersen J.V., Schousboe A., Wellendorph P. (2023). Astrocytes Regulate Inhibitory Neurotransmission through GABA Uptake, Metabolism, and Recycling. Essays Biochem..

[96] Hertz L. (2013). The Glutamate-Glutamine (GABA) Cycle: Importance of Late Postnatal Development and Potential Reciprocal Interactions between Biosynthesis and Degradation. Front. Endocrinol..

[97] Abdallah C.G., Jackowski A., Sato J.R., Mao X., Kang G., Cheema R., Coplan J.D., Mathew S.J., Shungu D.C. (2015). Prefrontal Cortical GABA Abnormalities Are Associated with Reduced Hippocampal Volume in Major Depressive Disorder. Eur. Neuropsychopharmacol..

[98] Fee C., Banasr M., Sibille E. (2017). Somatostatin-Positive Gamma-Aminobutyric Acid Interneuron Deficits in Depression: Cortical Microcircuit and Therapeutic Perspectives. Biol. Psychiatry.

[99] Prévot T., Sibille E. (2021). Altered GABA-Mediated Information Processing and Cognitive Dysfunctions in Depression and Other Brain Disorders. Mol. Psychiatry.

[100] Petty F., Schlesser M.A. (2002). Plasma GABA in Affective Illness. J. Affect. Disord..

[101] Gold B.I., Bowers M.B., Roth R.H., Sweeney D.W. (1980). GABA Levels in CSF of Patients with Psychiatric Disorders. Am. J. Psychiatry.

[102] Duman R.S., Sanacora G., Krystal J.H. (2019). Altered Connectivity in Depression: GABA and Glutamate Neurotransmitter Deficits and Reversal by Novel Treatments. Neuron.

[103] Gerner R.H., Hare T.A. (1981). CSF GABA in Normal Subjects and Patients with Depression, Schizophrenia, Mania, and Anorexia Nervosa. Am. J. Psychiatry.

[104] Zhao J., Bao A.M., Qi X.R., Kamphuis W., Luchetti S., Lou J.S., Swaab D.F. (2012). Gene Expression of GABA and Glutamate Pathway Markers in the Prefrontal Cortex of Non-Suicidal Elderly Depressed Patients. J. Affect. Disord..

[105] Allen N.J., Barres B.A. (2009). Neuroscience: Glia—More than Just Brain Glue. Nature.

[106] Somjen G.G. (1988). Nervenkitt: Notes on the History of the Concept of Neuroglia. Glia.

[107] Takahashi S. (2021). Neuroprotective Function of High Glycolytic Activity in Astrocytes: Common Roles in Stroke and Neurodegenerative Diseases. Int. J. Mol. Sci..

[108] Magistretti P.J., Pellerin L. (1999). Cellular Mechanisms of Brain Energy Metabolism and Their Relevance to Functional Brain Imaging. Philos. Trans. R. Soc. Lond. B Biol. Sci..

[109] Martinet J.L., Hardy P., Feline A., Huret J.D., Mazoyer B., Attar-Levy D., Pappata S., Syrota A. (1990). Left Prefrontal Glucose Hypometabolism in the Depressed State: A Confirmation. Am. J. Psychiatry.

[110] Nofzinger E.A., Nichols T.E., Meltzer C.C., Price J., Steppe D.A., Miewald J.M., Kupfer D.J., Moore R.Y. (1999). Changes in Forebrain Function from Waking to REM Sleep in Depression: Preliminary Analyses [of 18F]FDG PET Studies. Psychiatry Res. Neuroimaging.

[111] Hundal Ø. (2007). Major Depressive Disorder Viewed as a Dysfunction in Astroglial Bioenergetics. Med. Hypotheses.

[112] Gill T., Watling S.E., Richardson J.D., McCluskey T., Tong J., Meyer J.H., Warsh J., Jetly R., Hutchison M.G., Rhind S.G. (2022). Imaging of Astrocytes in Posttraumatic Stress Disorder: A PET Study with the Monoamine Oxidase B Radioligand [11C] SL25. 1188. Eur. Neuropsychopharmacol..

[113] Wingo T.S., Gerasimov E.S., Liu Y., Duong D.M., Vattathil S.M., Lori A., Gockley J., Breen M.S., Maihofer A.X., Nievergelt C.M. (2022). Integrating Human Brain Proteomes with Genome-Wide Association Data Implicates Novel Proteins in Post-Traumatic Stress Disorder. Mol. Psychiatry.

[114] Chatzinakos C., Pernia C.D., Morrison F.G., Iatrou A., McCullough K.M., Schuler H., Snijders C., Bajaj T., DiPietro C.P., Soliva Estruch M. (2023). Single-Nucleus Transcriptome Profiling of Dorsolateral Prefrontal Cortex: Mechanistic Roles for Neuronal Gene Expression, Including the 17q21.31 Locus, in PTSD Stress Response. Am. J. Psychiatry.

[115] Atrooz F., Alkadhi K.A., Salim S. (2021). Understanding Stress: Insights from Rodent Models. Curr. Res. Neurobiol..

[116] Campos A.C., Fogaça M.V., Aguiar D.C., Guimarães F.S. (2013). Animal Models of Anxiety Disorders and Stress. Braz. J. Psychiatry.

[117] Planchez B., Surget A., Belzung C. (2019). Animal Models of Major Depression: Drawbacks and Challenges. J. Neural. Transm..

[118] Banasr M., Duman R.S. (2008). Glial Loss in the Prefrontal Cortex Is Sufficient to Induce Depressive-like Behaviors. Biol. Psychiatry.

[119] Gosselin R.D., Gibney S., O’Malley D., Dinan T.G., Cryan J.F. (2009). Region Specific Decrease in Glial Fibrillary Acidic Protein Immunoreactivity in the Brain of a Rat Model of Depression. Neuroscience.

[120] Shilpa B.M., Bhagya V., Harish G., Srinivas Bharath M.M., Shankaranarayana Rao B.S. (2017). Environmental Enrichment Ameliorates Chronic Immobilisation Stress-Induced Spatial Learning Deficits and Restores the Expression of BDNF, VEGF, GFAP and Glucocorticoid Receptors. Prog. Neuropsychopharmacol. Biol. Psychiatry.

[121] Sántha P., Veszelka S., Hoyk Z., Mészáros M., Walter F.R., Tóth A.E., Kiss L., Kincses A., Oláh Z., Seprényi G. (2016). Restraint Stress-Induced Morphological Changes at the Blood-Brain Barrier in Adult Rats. Front. Mol. Neurosci..

[122] Tynan R.J., Beynon S.B., Hinwood M., Johnson S.J., Nilsson M., Woods J.J., Walker F.R. (2013). Chronic Stress-Induced Disruption of the Astrocyte Network Is Driven by Structural Atrophy and Not Loss of Astrocytes. Acta Neuropathol..

[123] Codeluppi S.A., Chatterjee D., Prevot T.D., Bansal Y., Misquitta K.A., Sibille E., Banasr M. (2021). Chronic Stress Alters Astrocyte Morphology in Mouse Prefrontal Cortex. Int. J. Neuropsychopharmacol..

[124] Czéh B., Simon M., Schmelting B., Hiemke C., Fuchs E. (2006). Astroglial Plasticity in the Hippocampus Is Affected by Chronic Psychosocial Stress and Concomitant Fluoxetine Treatment. Neuropsychopharmacology.

[125] Lou Y.X., Li J., Wang Z.Z., Xia C.Y., Chen N.H. (2018). Glucocorticoid Receptor Activation Induces Decrease of Hippocampal Astrocyte Number in Rats. Psychopharmacology.

[126] Araya-Callís C., Hiemke C., Abumaria N., Flugge G. (2012). Chronic Psychosocial Stress and Citalopram Modulate the Expression of the Glial Proteins GFAP and NDRG2 in the Hippocampus. Psychopharmacology.

[127] Liu W.X., Wang J., Xie Z.M., Xu N., Zhang G.F., Jia M., Zhou Z.Q., Hashimoto K., Yang J.J. (2016). Regulation of Glutamate Transporter 1 via BDNF-TrkB Signaling Plays a Role in the Anti-Apoptotic and Antidepressant Effects of Ketamine in Chronic Unpredictable Stress Model of Depression. Psychopharmacology.

[128] Yang C.R., Zhang Z.G., Bai Y.Y., Zhou H.F., Zhou L., Ruan C.S., Li F., Li C.Q., Zheng H.Y., Shen L.J. (2014). Foraging Activity Is Reduced in a Mouse Model of Depression. Neurotox. Res..

[129] Ye Y., Wang G., Wang H., Wang X. (2011). Brain-Derived Neurotrophic Factor (BDNF) Infusion Restored Astrocytic Plasticity in the Hippocampus of a Rat Model of Depression. Neurosci. Lett..

[130] Naskar S., Chattarji S. (2019). Stress Elicits Contrasting Effects on the Structure and Number of Astrocytes in the Amygdala versus Hippocampus. eNeuro.

[131] Naskar S., Datta S., Chattarji S. (2022). Riluzole Prevents Stress-Induced Spine Plasticity in the Hippocampus but Mimics It in the Amygdala. Neurobiol. Stress..

[132] Kwon M.S., Seo Y.J., Lee J.K., Lee H.K., Jung J.S., Jang J.E., Park S.H., Suh H.W. (2008). The Repeated Immobilization Stress Increases IL-1beta Immunoreactivities in Only Neuron, but Not Astrocyte or Microglia in Hippocampal CA1 Region, Striatum and Paraventricular Nucleus. Neurosci. Lett..

[133] Dong L., Wang S., Li Y., Zhao Z., Shen Y., Liu L., Xu G., Ma C., Li S., Zhang X. (2017). RU486 Reverses Emotional Disorders by Influencing Astrocytes and Endoplasmic Reticulum Stress in Chronic Restraint Stress Challenged Rats. Cell Physiol. Biochem..

[134] Du Preez A., Onorato D., Eiben I., Musaelyan K., Egeland M., Zunszain P.A., Fernandes C., Thuret S., Pariante C.M. (2021). Chronic Stress Followed by Social Isolation Promotes Depressive-like Behaviour, Alters Microglial and Astrocyte Biology and Reduces Hippocampal Neurogenesis in Male Mice. Brain Behav. Immun..

[135] Simard S., Coppola G., Rudyk C.A., Hayley S., McQuaid R.J., Salmaso N. (2018). Profiling Changes in Cortical Astroglial Cells Following Chronic Stress. Neuropsychopharmacology.

[136] Imbe H., Kimura A., Donishi T., Kaneoke Y. (2012). Chronic Restraint Stress Decreases Glial Fibrillary Acidic Protein and Glutamate Transporter in the Periaqueductal Gray Matter. Neuroscience.

[137] Baek J.H., Vignesh A., Son H., Lee D.H., Roh G.S., Kang S.S., Cho G.J., Choi W.S., Kim H.J. (2019). Glutamine Supplementation Ameliorates Chronic Stress-Induced Reductions in Glutamate and Glutamine Transporters in the Mouse Prefrontal Cortex. Exp. Neurobiol..

[138] Veeraiah P., Noronha J.M., Maitra S., Bagga P., Khandelwal N., Chakravarty S., Kumar A., Patel A.B. (2014). Dysfunctional Glutamatergic and γ-Aminobutyric Acidergic Activities in Prefrontal Cortex of Mice in Social Defeat Model of Depression. Biol. Psychiatry.

[139] Chen J.X., Yao L.H., Xu B.B., Qian K., Wang H.L., Liu Z.C., Wang X.P., Wang G.H. (2014). Glutamate Transporter 1-Mediated Antidepressant-like Effect in a Rat Model of Chronic Unpredictable Stress. J. Huazhong Univ. Sci. Technolog. Med. Sci..

[140] Zink M., Vollmayr B., Gebicke-Haerter P.J., Henn F.A. (2010). Reduced Expression of Glutamate Transporters VGluT1, EAAT2 and EAAT4 in Learned Helpless Rats, an Animal Model of Depression. Neuropharmacology.

[141] Reagan L.P., Rosell D.R., Wood G.E., Spedding M., Muñoz C., Rothstein J., McEwen B.S. (2004). Chronic Restraint Stress Up-Regulates GLT-1 MRNA and Protein Expression in the Rat Hippocampus: Reversal by Tianeptine. Proc. Natl. Acad. Sci. USA.

[142] Raudensky J., Yamamoto B.K. (2007). Effects of Chronic Unpredictable Stress and Methamphetamine on Hippocampal Glutamate Function. Brain Res..

[143] Liu F., Wu J., Gong Y., Wang P., Zhu L., Tong L., Chen X., Ling Y., Huang C. (2017). Harmine Produces Antidepressant-like Effects via Restoration of Astrocytic Functions. Prog. Neuropsychopharmacol. Biol. Psychiatry.

[144] Miguel-Hidalgo J.J., Moulana M., Deloach P.H., Rajkowska G. (2018). Chronic Unpredictable Stress Reduces Immunostaining for Connexins 43 and 30 and Myelin Basic Protein in the Rat Prelimbic and Orbitofrontal Cortices. Chronic Stress.

[145] Sun J.D., Liu Y., Yuan Y.H., Li J., Chen N.H. (2012). Gap Junction Dysfunction in the Prefrontal Cortex Induces Depressive-like Behaviors in Rats. Neuropsychopharmacology.

[146] Cao X., Li L.P., Wang Q., Wu Q., Hu H.H., Zhang M., Fang Y.Y., Zhang J., Li S.J., Xiong W.C. (2013). Astrocyte-Derived ATP Modulates Depressive-like Behaviors. Nat. Med..

[147] Srivastava I., Vazquez-Juarez E., Henning L., Gómez-Galán M., Lindskog M. (2020). Blocking Astrocytic GABA Restores Synaptic Plasticity in Prefrontal Cortex of Rat Model of Depression. Cells.

[148] Flandreau E.I., Toth M. (2018). Animal Models of PTSD: A Critical Review. Curr. Top. Behav. Neurosci..

[149] Perez-Urrutia N., Mendoza C., Alvarez-Ricartes N., Oliveros-Matus P., Echeverria F., Grizzell J.A., Barreto G.E., Iarkov A., Echeverria V. (2017). Intranasal Cotinine Improves Memory, and Reduces Depressive-like Behavior, and GFAP+ Cells Loss Induced by Restraint Stress in Mice. Exp. Neurol..

[150] Feng D., Guo B., Liu G., Wang B., Wang W., Gao G., Qin H., Wu S. (2015). FGF2 Alleviates PTSD Symptoms in Rats by Restoring GLAST Function in Astrocytes via the JAK/STAT Pathway. Eur. Neuropsychopharmacol..

[151] Saur L., Baptista P.P.A., Bagatini P.B., Neves L.T., de Oliveira R.M., Vaz S.P., Ferreira K., Machado S.A., Mestriner R.G., Xavier L.L. (2016). Experimental Post-Traumatic Stress Disorder Decreases Astrocyte Density and Changes Astrocytic Polarity in the CA1 Hippocampus of Male Rats. Neurochem. Res..

[152] Han F., Xiao B., Wen L. (2015). Loss of Glial Cells of the Hippocampus in a Rat Model of Post-Traumatic Stress Disorder. Neurochem. Res..

[153] Li H., Tofigh A.M., Amirfakhraei A., Chen X., Tajik M., Xu D., Motevalli S. (2022). Modulation of Astrocyte Activity and Improvement of Oxidative Stress through Blockage of NO/NMDAR Pathway Improve Posttraumatic Stress Disorder (PTSD)-like Behavior Induced by Social Isolation Stress. Brain Behav..

[154] Xia L., Zhai M., Wang L., Miao D., Zhu X., Wang W. (2013). FGF2 Blocks PTSD Symptoms via an Astrocyte-Based Mechanism. Behav. Brain Res..

[155] Iwata M., Shirayama Y., Ishida H., Hazama G.I., Nakagome K. (2011). Hippocampal Astrocytes Are Necessary for Antidepressant Treatment of Learned Helplessness Rats. Hippocampus.

[156] Brockett A.T., Kane G.A., Monari P.K., Briones B.A., Vigneron P.A., Barber G.A., Bermudez A., Dieffenbach U., Kloth A.D., Buschman T.J. (2018). Evidence Supporting a Role for Astrocytes in the Regulation of Cognitive Flexibility and Neuronal Oscillations through the Ca^2+^ Binding Protein S100β. PLoS ONE.

[157] Etiévant A., Oosterhof C., Bétry C., Abrial E., Novo-Perez M., Rovera R., Scarna H., Devader C., Mazella J., Wegener G. (2015). Astroglial Control of the Antidepressant-Like Effects of Prefrontal Cortex Deep Brain Stimulation. EBioMedicine.

[158] Domin H., Szewczyk B., Woźniak M., Wawrzak-Wleciał A., Śmiałowska M. (2014). Antidepressant-like Effect of the MGluR5 Antagonist MTEP in an Astroglial Degeneration Model of Depression. Behav. Brain Res..

[159] David J., Gormley S., McIntosh A.L., Kebede V., Thuery G., Varidaki A., Coffey E.T., Harkin A. (2019). L-Alpha-Amino Adipic Acid Provokes Depression-like Behaviour and a Stress Related Increase in Dendritic Spine Density in the Pre-Limbic Cortex and Hippocampus in Rodents. Behav. Brain Res..

[160] Kim Y., Lee H.-Y., Choi Y.-J., Cho S.-H. (2020). Antidepressant Effects of Ginsenoside Rf on Behavioral Change in the Glial Degeneration Model of Depression by Reversing Glial Loss. J. Ginseng Res..

[161] Choi Y., Kim Y., Lee H.Y., Cho S.H. (2021). Tetragonia Tetragonioides Relieves Depressive-Like Behavior through the Restoration of Glial Loss in the Prefrontal Cortex. Evid. Based Complement. Alternat Med..

[162] Codeluppi S.A., Xu M., Bansal Y., Lepack A.E., Duric V., Chow M., Muir J., Bagot R.C., Licznerski P., Wilber S.L. (2023). Prefrontal Cortex Astroglia Modulate Anhedonia-like Behavior. Mol. Psychiatry.

[163] Bechtholt-Gompf A.J., Walther H.V., Adams M.A., Carlezon W.A., Ngür D., Cohen B.M. (2010). Blockade of Astrocytic Glutamate Uptake in Rats Induces Signs of Anhedonia and Impaired Spatial Memory. Neuropsychopharmacology.

[164] Tian S.W., Yu X.D., Cen L., Xiao Z.Y. (2019). Glutamate Transporter GLT1 Inhibitor Dihydrokainic Acid Impairs Novel Object Recognition Memory Performance in Mice. Physiol. Behav..

[165] Saitoh A., Soda A., Kayashima S., Yoshizawa K., Oka J.I., Nagase H., Yamada M. (2018). A Delta Opioid Receptor Agonist, KNT-127, in the Prelimbic Medial Prefrontal Cortex Attenuates Glial Glutamate Transporter Blocker-Induced Anxiety-like Behavior in Mice. J. Pharmacol. Sci..

[166] Fullana M.N., Ruiz-Bronchal E., Ferrés-Coy A., Juárez-Escoto E., Artigas F., Bortolozzi A. (2019). Regionally Selective Knockdown of Astroglial Glutamate Transporters in Infralimbic Cortex Induces a Depressive Phenotype in Mice. Glia.

[167] Gasull-Camós J., Arrés-Gatius M.T., Artigas F., Castañé A. (2017). Glial GLT-1 Blockade in Infralimbic Cortex as a New Strategy to Evoke Rapid Antidepressant-like Effects in Rats. Transl. Psychiatry.

[168] Gasull-Camós J., Martínez-Torres S., Tarrés-Gatius M., Ozaita A., Artigas F., Castañé A. (2018). Serotonergic Mechanisms Involved in Antidepressant-like Responses Evoked by GLT-1 Blockade in Rat Infralimbic Cortex. Neuropharmacology.

[169] Liu X., Guo H., Sayed M.D.S., Lu Y., Yang T., Zhou D., Chen Z., Wang H., Wang C., Xu J. (2016). CAMP/PKA/CREB/GLT1 Signaling Involved in the Antidepressant-like Effects of Phosphodiesterase 4D Inhibitor (GEBR-7b) in Rats. Neuropsychiatr. Dis. Treat..

[170] Gasull-Camós J., Soto-Montenegro M.L., Casquero-Veiga M., Desco M., Artigas F., Castañé A. (2017). Differential Patterns of Subcortical Activity Evoked by Glial GLT-1 Blockade in Prelimbic and Infralimbic Cortex: Relationship to Antidepressant-Like Effects in Rats. Int. J. Neuropsychopharmacol..

[171] Cui Y., Yang Y., Ni Z., Dong Y., Cai G., Foncelle A., Ma S., Sang K., Tang S., Li Y. (2018). Astroglial Kir4.1 in the Lateral Habenula Drives Neuronal Bursts in Depression. Nature.

[172] Wu X., Li L., Zhou B., Wang J., Shao W. (2023). Connexin 43 Regulates Astrocyte Dysfunction and Cognitive Deficits in Early Life Stress-Treated Mice. Exp. Brain Res..

[173] Papouin T., Dunphy J., Tolman M., Foley J.C., Haydon P.G. (2017). Astrocytic Control of Synaptic Function. Philos. Trans. R. Soc. Lond. B Biol. Sci..

[174] Araque A. (2008). Astrocytes Process Synaptic Information. Neuron Glia Biol..

[175] Cho W.H., Noh K., Lee B.H., Barcelon E., Jun S.B., Park H.Y., Lee S.J. (2022). Hippocampal Astrocytes Modulate Anxiety-like Behavior. Nat. Commun..

[176] Jones M.E., Paniccia J.E., Lebonville C.L., Reissner K.J., Lysle D.T. (2018). Chemogenetic Manipulation of Dorsal Hippocampal Astrocytes Protects Against the Development of Stress-Enhanced Fear Learning. Neuroscience.

[177] Novakovic M.M., Korshunov K.S., Grant R.A., Martin M.E., Valencia H.A., Budinger G.R.S., Radulovic J., Prakriya M. (2023). Astrocyte Reactivity and Inflammation-Induced Depression-like Behaviors Are Regulated by Orai1 Calcium Channels. Nat. Commun..

[178] Srinivasan R., Huang B.S., Venugopal S., Johnston A.D., Chai H., Zeng H., Golshani P., Khakh B.S. (2015). Ca(2+) Signaling in Astrocytes from Ip3r2(−/−) Mice in Brain Slices and during Startle Responses In Vivo. Nat. Neurosci..

[179] Petravicz J., Boyt K.M., McCarthy K.D. (2014). Astrocyte IP3R2-Dependent Ca(2+) Signaling Is Not a Major Modulator of Neuronal Pathways Governing Behavior. Front. Behav. Neurosci..

[180] Delcourte S., Bouloufa A., Rovera R., Bétry C., Abrial E., Dkhissi-Benyahya O., Heinrich C., Marcy G., Raineteau O., Haddjeri N. (2023). Chemogenetic Activation of Prefrontal Astroglia Enhances Recognition Memory Performance in Rat. Biomed. Pharmacother..

[181] Adamsky A., Kol A., Kreisel T., Doron A., Ozeri-Engelhard N., Melcer T., Refaeli R., Horn H., Regev L., Groysman M. (2018). Astrocytic Activation Generates De Novo Neuronal Potentiation and Memory Enhancement. Cell.

[182] Adamsky A., Goshen I. (2018). Astrocytes in Memory Function: Pioneering Findings and Future Directions. Neuroscience.

[183] Kol A., Adamsky A., Groysman M., Kreisel T., London M., Goshen I. (2020). Astrocytes Contribute to Remote Memory Formation by Modulating Hippocampal-Cortical Communication during Learning. Nat. Neurosci..

[184] Mederos S., Hernández-Vivanco A., Ramírez-Franco J., Martín-Fernández M., Navarrete M., Yang A., Boyden E.S., Perea G. (2019). Melanopsin for Precise Optogenetic Activation of Astrocyte-Neuron Networks. Glia.

[185] Suthard R.L., Senne R.A., Buzharsky M.D., Diep A.H., Pyo A.Y., Ramirez S. (2024). Engram Reactivation Mimics Cellular Signatures of Fear. Cell Rep..

[186] Suthard R.L., Senne R.A., Buzharsky M.D., Pyo A.Y., Dorst K.E., Diep A.H., Cole R.H., Ramirez S. (2023). Basolateral Amygdala Astrocytes Are Engaged by the Acquisition and Expression of a Contextual Fear Memory. J. Neurosci..

[187] Lei Z., Xie L., Li C.H., Lam Y.Y., Ramkrishnan A.S., Fu Z., Zeng X., Liu S., Iqbal Z., Li Y. (2022). Chemogenetic Activation of Astrocytes in the Basolateral Amygdala Contributes to Fear Memory Formation by Modulating the Amygdala-Prefrontal Cortex Communication. Int. J. Mol. Sci..

[188] González-Arias C., Sánchez-Ruiz A., Esparza J., Sánchez-Puelles C., Arancibia L., Ramírez-Franco J., Gobbo D., Kirchhoff F., Perea G. (2023). Dysfunctional Serotonergic Neuron-Astrocyte Signaling in Depressive-like States. Mol. Psychiatry.

[189] Erickson E.K., DaCosta A.J., Mason S.C., Blednov Y.A., Mayfield R.D., Harris R.A. (2021). Cortical Astrocytes Regulate Ethanol Consumption and Intoxication in Mice. Neuropsychopharmacology.

[190] Boden J.M., Fergusson D.M. (2011). Alcohol and Depression. Addiction.

[191] Grant B.F., Goldstein R.B., Saha T.D., Patricia Chou S., Jung J., Zhang H., Pickering R.P., June Ruan W., Smith S.M., Huang B. (2015). Epidemiology of DSM-5 Alcohol Use Disorder Results from the National Epidemiologic Survey on Alcohol and Related Conditions III. JAMA Psychiatry.

[192] Miguel-Hidalgo J., Shoyama Y., Wanzo V. (2009). Infusion of Gliotoxins or a Gap Junction Blocker in the Prelimbic Cortex Increases Alcohol Preference in Wistar Rats. J. Psychopharmacol..

[193] Liu Y., Chen L., Lin L., Xu C., Xiong Y., Qiu H., Li X., Li S., Cao H. (2024). Unveiling the Hidden Pathways: Exploring Astrocytes as a Key Target for Depression Therapy. J. Psychiatr. Res..

[194] Peng L., Verkhratsky A., Gu L., Li B. (2015). Targeting Astrocytes in Major Depression. Expert. Rev. Neurother..

[195] Manev H., Uz T., Manev R. (2003). Glia as a Putative Target for Antidepressant Treatments. J. Affect. Disord..

[196] Quesseveur G., Gardier A., Guiard B. (2013). The Monoaminergic Tripartite Synapse: A Putative Target for Currently Available Antidepressant Drugs. Curr. Drug Targets.

[197] Frizzo M.E., Ohno Y. (2021). Perisynaptic Astrocytes as a Potential Target for Novel Antidepressant Drugs. J. Pharmacol. Sci..

[198] Marathe S.V., D’almeida P.L., Virmani G., Bathini P., Alberi L. (2018). Effects of Monoamines and Antidepressants on Astrocyte Physiology: Implications for Monoamine Hypothesis of Depression. J. Exp. Neurosci..

[199] Banasr M., Dwyer J.M., Duman R.S. (2011). Cell Atrophy and Loss in Depression: Reversal by Antidepressant Treatment. Curr. Opin. Cell Biol..

[200] Duman C.H., Duman R.S. (2015). Spine Synapse Remodeling in the Pathophysiology and Treatment of Depression. Neurosci. Lett..

[201] Harmer C.J., Duman R.S., Cowen P.J. (2017). How Do Antidepressants Work? New Perspectives for Refining Future Treatment Approaches. Lancet Psychiatry.

[202] Allaman I., Fiumelli H., Magistretti P.J., Martin J.L. (2011). Fluoxetine Regulates the Expression of Neurotrophic/Growth Factors and Glucose Metabolism in Astrocytes. Psychopharmacology.

[203] Hisaoka-Nakashima K., Kajitani N., Kaneko M., Shigetou T., Kasai M., Matsumoto C., Yokoe T., Azuma H., Takebayashi M., Morioka N. (2016). Amitriptyline Induces Brain-Derived Neurotrophic Factor (BDNF) MRNA Expression through ERK-Dependent Modulation of Multiple BDNF MRNA Variants in Primary Cultured Rat Cortical Astrocytes and Microglia. Brain Res..

[204] Kittel-Schneider S., Kenis G., Schek J., van den Hove D., Prickaerts J., Lesch K.P., Steinbusch H., Reif A. (2012). Expression of Monoamine Transporters, Nitric Oxide Synthase 3, and Neurotrophin Genes in Antidepressant-Stimulated Astrocytes. Front. Psychiatry.

[205] Jeanson T., Pondaven A., Ezan P., Mouthon F., Charvériat M., Giaume C. (2016). Antidepressants Impact Connexin 43 Channel Functions in Astrocytes. Front. Cell Neurosci..

[206] Kinoshita M., Hirayama Y., Fujishita K., Shibata K., Shinozaki Y., Shigetomi E., Takeda A., Le H.P.N., Hayashi H., Hiasa M. (2018). Anti-Depressant Fluoxetine Reveals Its Therapeutic Effect Via Astrocytes. EBioMedicine.

[207] Kong H., Sha L.L., Fan Y., Xiao M., Ding J.H., Wu J., Hu G. (2009). Requirement of AQP4 for Antidepressive Efficiency of Fluoxetine: Implication in Adult Hippocampal Neurogenesis. Neuropsychopharmacology.

[208] Xia C.Y., Zhang N.N., Jiang H., Lou Y.X., Ren Q., Zhang X.L., Yang P.F., Shao Q.H., Zhu H.Y., Wan J.F. (2023). Gap Junction Is Essential for the Antidepressant Effects of Fluoxetine. J. Pharm. Pharmacol..

[209] Fullana M.N., Covelo A., Bortolozzi A., Araque A., Artigas F. (2019). In Vivo Knockdown of Astroglial Glutamate Transporters GLT-1 and GLAST Increases Excitatory Neurotransmission in Mouse Infralimbic Cortex: Relevance for Depressive-like Phenotypes. Eur. Neuropsychopharmacol..

[210] Fullana M.N., Paz V., Artigas F., Bortolozzi A. (2022). Ketamine Triggers Rapid Antidepressant Effects by Modulating Synaptic Plasticity in a New Depressive-like Mouse Model Based on Astrocyte Glutamate Transporter GLT-1 Knockdown in Infralimbic Cortex. Rev. Psiquiatr. Salud. Ment. (Engl. Ed.).

[211] Stenovec M. (2021). Ketamine Alters Functional Plasticity of Astroglia: An Implication for Antidepressant Effect. Life.

[212] Stenovec M., Li B., Verkhratsky A., Zorec R. (2020). Astrocytes in Rapid Ketamine Antidepressant Action. Neuropharmacology.

[213] Pham T.H., Defaix C., Nguyen T.M.L., Mendez-David I., Tritschler L., David D.J., Gardier A.M. (2020). Cortical and Raphe GABAA, AMPA Receptors and Glial GLT-1 Glutamate Transporter Contribute to the Sustained Antidepressant Activity of Ketamine. Pharmacol. Biochem. Behav..

[214] Li J.F., Hu W.Y., Chang H.X., Bao J.H., Kong X.X., Ma H., Li Y.F. (2023). Astrocytes Underlie a Faster-Onset Antidepressant Effect of Hypidone Hydrochloride (YL-0919). Front. Pharmacol..

[215] Ma X., Yang S., Zhang Z., Liu L., Shi W., Yang S., Li S., Cai X., Zhou Q. (2022). Rapid and Sustained Restoration of Astrocytic Functions by Ketamine in Depression Model Mice. Biochem. Biophys. Res. Commun..

[216] Mineur Y.S., Picciotto M.R., Sanacora G. (2007). Antidepressant-like Effects of Ceftriaxone in Male C57BL/6J Mice. Biol. Psychiatry.

[217] Banasr M., Chowdhury G.M.I., Terwilliger R., Newton S.S., Duman R.S., Behar K.L., Sanacora G. (2010). Glial Pathology in an Animal Model of Depression: Reversal of Stress-Induced Cellular, Metabolic and Behavioral Deficits by the Glutamate-Modulating Drug Riluzole. Mol. Psychiatry.

[218] Bansal Y., Fee C., Misquitta K.A., Codeluppi S.A., Sibille E., Berman R.M., Coric V., Sanacora G., Banasr M. (2023). Prophylactic Efficacy of Riluzole against Anxiety- and Depressive-Like Behaviors in Two Rodent Stress Models. Complex. Psychiatry.

[219] Almeida R.F., Nonose Y., Ganzella M., Loureiro S.O., Rocha A., Machado D.G., Bellaver B., Fontella F.U., Leffa D.T., Pettenuzzo L.F. (2021). Antidepressant-Like Effects of Chronic Guanosine in the Olfactory Bulbectomy Mouse Model. Front. Psychiatry.

[220] Rosa P.B., Bettio L.E.B., Neis V.B., Moretti M., Kaufmann F.N., Tavares M.K., Werle I., Dalsenter Y., Platt N., Rosado A.F. (2021). Antidepressant-like Effect of Guanosine Involves Activation of AMPA Receptor and BDNF/TrkB Signaling. Purinergic Signal.

[221] Fortunato J.J., Réus G.Z., Kirsch T.R., Stringari R.B., Fries G.R., Kapczinski F., Hallak J.E., Zuardi A.W., Crippa J.A., Quevedo J. (2010). Effects of Beta-Carboline Harmine on Behavioral and Physiological Parameters Observed in the Chronic Mild Stress Model: Further Evidence of Antidepressant Properties. Brain Res. Bull..

[222] Fortunato J.J., Réus G.Z., Kirsch T.R., Stringari R.B., Fries G.R., Kapczinski F., Hallak J.E., Zuardi A.W., Crippa J.A., Quevedo J. (2010). Chronic Administration of Harmine Elicits Antidepressant-like Effects and Increases BDNF Levels in Rat Hippocampus. J. Neural. Transm..

[223] Gourley S.L., Espitia J.W., Sanacora G., Taylor J.R. (2012). Antidepressant-like Properties of Oral Riluzole and Utility of Incentive Disengagement Models of Depression in Mice. Psychopharmacology.

[224] Kaur B., Prakash A. (2017). Ceftriaxone Attenuates Glutamate-Mediated Neuro-Inflammation and Restores BDNF in MPTP Model of Parkinson’s Disease in Rats. Pathophysiology.

[225] Katoh-Semba R., Asano T., Ueda H., Morishita R., Takeuchi I.K., Inaguma Y., Kato K. (2002). Riluzole Enhances Expression of Brain-Derived Neurotrophic Factor with Consequent Proliferation of Granule Precursor Cells in the Rat Hippocampus. FASEB J..

[226] Li S.P., Wang Y.W., Qi S.L., Zhang Y.P., Deng G., Ding W.Z., Ma C., Lin Q.Y., Guan H.D., Liu W. (2018). Analogous β-Carboline Alkaloids Harmaline and Harmine Ameliorate Scopolamine-Induced Cognition Dysfunction by Attenuating Acetylcholinesterase Activity, Oxidative Stress, and Inflammation in Mice. Front. Pharmacol..

[227] Liu W.Z., Huang B.W., You W.J., Hu P., Wang X.H., Zhang J.Y., Xu X.B., Zhang Z.Y., Pan B.X., Zhang W.H. (2018). Harmine Enhances GABAergic Transmission onto Basoamygdala Projection Neurons in Mice. Brain Res. Bull..

[228] Feng D., Wang W., Dong Y., Wu L., Huang J., Ma Y., Zhang Z., Wu S., Gao G., Qin H. (2014). Ceftriaxone Alleviates Early Brain Injury after Subarachnoid Hemorrhage by Increasing Excitatory Amino Acid Transporter 2 Expression via the PI3K/Akt/NF-ΚB Signaling Pathway. Neuroscience.

[229] Camargo A., Dalmagro A.P., Delanogare E., Fraga D.B., Wolin I.A.V., Zeni A.L.B., Brocardo P.S., Rodrigues A.L.S. (2022). Guanosine Boosts the Fast, but Not Sustained, Antidepressant-like and pro-Synaptogenic Effects of Ketamine by Stimulating MTORC1-Driven Signaling Pathway. Eur. Neuropsychopharmacol..

[230] Salardini E., Zeinoddini A., Mohammadinejad P., Khodaie-Ardakani M.R., Zahraei N., Zeinoddini A., Akhondzadeh S. (2016). Riluzole Combination Therapy for Moderate-to-Severe Major Depressive Disorder: A Randomized, Double-Blind, Placebo-Controlled Trial. J. Psychiatr. Res..

[231] Mathew S.J., Gueorguieva R., Brandt C., Fava M., Sanacora G. (2017). A Randomized, Double-Blind, Placebo-Controlled, Sequential Parallel Comparison Design Trial of Adjunctive Riluzole for Treatment-Resistant Major Depressive Disorder. Neuropsychopharmacology.

[232] Zarate C.A., Payne J.L., Quiroz J., Sporn J., Denicoff K.K., Luckenbaugh D., Charney D.S., Manji H.K. (2004). An Open-Label Trial of Riluzole in Patients with Treatment-Resistant Major Depression. Am. J. Psychiatry.

[233] Sanacora G., Kendell S.F., Levin Y., Simen A.A., Fenton L.R., Coric V., Krystal J.H. (2007). Preliminary Evidence of Riluzole Efficacy in Antidepressant-Treated Patients with Residual Depressive Symptoms. Biol. Psychiatry.

[234] Spangler P.T., West J.C., Dempsey C.L., Possemato K., Bartolanzo D., Aliaga P., Zarate C., Vythilingam M., Benedek D.M. (2020). Randomized Controlled Trial of Riluzole Augmentation for Posttraumatic Stress Disorder: Efficacy of a Glutamatergic Modulator for Antidepressant-Resistant Symptoms. J. Clin. Psychiatry.

[235] Kang S., Li J., Bekker A., Ye J.H. (2018). Rescue of Glutamate Transport in the Lateral Habenula Alleviates Depression- and Anxiety-like Behaviors in Ethanol-Withdrawn Rats. Neuropharmacology.

[236] de Almeida R.F., Pocharski C.B., Rodrigues A.L.S., Elisabetsky E., Souza D.O. (2020). Guanosine Fast Onset Antidepressant-like Effects in the Olfactory Bulbectomy Mice Model. Sci. Rep..

[237] Fontana A.C.K. (2015). Current Approaches to Enhance Glutamate Transporter Function and Expression. J. Neurochem..

